# Generalized Resistance Deficits in inmates with hypertension: missing resources that limit health

**DOI:** 10.1590/0034-7167-2023-0246

**Published:** 2024-05-13

**Authors:** Marta Cossetin Costa, Maria de Fátima Mantovani, Fernanda Moura D’Almeida Miranda, Ivonete Teresinha Schülter Buss Heidemann, Aida Maris Peres

**Affiliations:** I Universidade Federal do Paraná. Curitiba, Paraná, Brazil; II Universidade Federal de Santa Catarina. Florianópolis, Brazil

**Keywords:** Prisoners, Prisons, Health Promotion, Salutogenesis, Adult Health, Personas Privadas de Libertad, Cárcel, Promoción de la Salud, Salutogénesis, Salud del Adulto, Pessoas Privadas de Liberdade, Prisão, Promoção da Saúde, Salutogênese, Saúde do Adulto

## Abstract

**Objective:**

to understand the Generalized Resistance Deficits of people deprived of liberty with hypertension in a Brazilian prison unit.

**Method:**

qualitative research, anchored in Salutogenesis, carried out with 38 people with hypertension from a Brazilian prison unit, from February to July 2022, with a semi-structured interview with open-ended questions, whose analysis was thematic, explaining the limitations to health in prison.

**Results:**

13 Generalized Resistance Deficits were reported, mostly related to the prison environment and, to a lesser extent, to the social group and the individual, respectively. Living in prison for people with hypertension implies living with a high number of Generalized Resistance Deficits, accentuating the movement towards the disease pole.

**Final considerations:**

knowing Generalized Resistance Deficits allows directing health promotion to support the use of available Generalized Resistance Resources and contributes to the expansion of intersectoral policies.

## RESUMO

**Objetivo:**

apreender os Déficits Generalizados de Resistência de pessoas privadas de liberdade com hipertensão arterial sistêmica de uma unidade prisional brasileira.

**Método:**

pesquisa qualitativa, ancorada na Salutogênese, realizada com 38 pessoas com hipertensão arterial sistêmica de uma unidade prisional brasileira, de fevereiro a julho de 2022, com entrevista semiestruturada de questões abertas, cuja análise foi temática, explicitando os limitantes à saúde na prisão.

**Resultados:**

foram relatados 13 Déficits Generalizados de Resistência, relacionando-se em maioria ao ambiente prisional e, em menor proporção, ao grupo social e ao indivíduo, respectivamente. O viver na prisão para pessoas com hipertensão arterial sistêmica implica conviver com elevado número de Déficits Generalizados de Resistência, acentuando o movimento em direção ao polo doença.

**Considerações finais:**

conhecer os Déficits Generalizados de Resistência permite direcionar a promoção da saúde para apoiar o uso dos Recursos Generalizados de Resistência disponíveis e contribui com a ampliação de políticas intersetoriais.

## RESUMEN

**Objetivo:**

comprender los Déficits de Resistencia Generalizados de personas privadas de libertad con hipertensión arterial sistémica en una unidad penitenciaria brasileña.

**Método:**

investigación cualitativa, anclada en la Salutogénesis, realizada con 38 personas con hipertensión arterial sistémica de una unidad penitenciaria brasileña, de febrero a julio de 2022, con entrevista semiestructurada con preguntas abiertas, cuyo análisis fue temático, explicando las limitaciones a la salud en prisión.

**Resultados:**

se reportaron 13 Déficits de Resistencia Generalizados, en su mayoría relacionados con el ambiente penitenciario y, en menor medida, con el grupo social y el individuo, respectivamente. Vivir en prisión para personas con hipertensión arterial sistémica implica vivir con un elevado número de Déficits de Resistencia Generalizados, acentuando el movimiento hacia el polo de la enfermedad.

**Consideraciones finales:**

conocer los Déficits de Resistencia Generalizada permite orientar la promoción de la salud para apoyar el uso de los Recursos de Resistencia Generalizada disponibles y contribuye a la ampliación de políticas intersectoriales.

## INTRODUCTION

People deprived of liberty (PDL) have a greater burden of physical and mental illness and risk of becoming ill when compared to the community outside prison, which is related to living conditions prior to prison, such as poverty, low education, limited access health, drug and alcohol use and trauma; situations that can be perpetrated and/or intensified in the prison environment. It is noted that health in prisons is understood as a fundamental human right and an element of social justice, and health services are components of primary care and constitute points of the Health Care Network, and must act within the scope of health promotion, prevention and recovery, including acute and chronic diseases^(1-3)^.

It is considered that the approach that guides this study is the Salutogenic Theory, whose focus is on what generates health, i.e., it focuses on understanding how people remain healthy despite stressors, as they are located at the healthy pole of the health-disease continuum. Thus, health or illness conditions depend on the adequate management of subjects’ tensions^(4)^.

From this perspective, the salutogenic approach to health in prisons emerges when prison health begins to be understood as a component of primary care in general, whose care must be equivalent to that outside the walls. Thus, the salutogenic model in prisons aims to implement public health strategies that allow creating health in such a context, with health promotion being the target, since the aim is to develop healthy correctional policies that provide opportunities for PDL and employees to access resources to improve the health of these inmates. It is worth highlighting that, to build healthier spaces, listening to PDL’s and employees’ opinions is essential so that strategies meet their needs^(3)^.

Furthermore, the approach to chronic non-communicable diseases (NCDs) is also fundamental in this context, considering that they account for 54.7% of total deaths in Brazil^(5)^. Cardiovascular diseases (CVD) were the main causes of death also among incarcerated and recently released people in the United States of America, with 89% of deaths related to NCDs^(6-7)^. Among CVDs, hypertension affects the general population with a percentage of 26.3%^(4)^ and, in a survey of 1,327 female PDL, found rates of 24.4%^(2)^. Therefore, controlling blood pressure in prisons is essential to avoid complications arising from it.

Furthermore, the prison context is also a setting for health promotion, and to this end, the salutogenic approach allows a focus on people’s health and lives instead of disease. The model’s central concepts are sense of coherence (SOC) and Generalized Resources of Resistance (GRRs). SOC consists of a global orientation to observe the world, in the sense of demonstrating belief in the possibility of overcoming the challenge so that the person believes in their ability to overcome them, such as understanding that they have the resources to meet the challenges stimuli and is motivated to deal with the situation. GRRs are any phenomena that are effective in combating stressors. Both elements, SOC and GRRs, have a reciprocal relationship, i.e., GRRs promote strong SOC and, in turn, this helps in mobilizing resources to manage tensions. Therefore, strong SOC facilitates the person’s movement towards health, considered a predictor of health^(8-9)^.

Generalized Resistance Deficits (GRDs) are also components of the Salutogenic Model, and are described as stressors or as the absence of resources, contributing to increasing chaos and disorder in people’s lives. In this regard, it is the negative experiences that favor the person to migrate to the disease pole on the health-disease continuum, which weakens SOC^(8,10-11)^.

The reciprocity between SOC and GRDs is asserted when they become chronic in a person’s life, which can contribute to failure to manage tensions effectively, leading subjects to the disease pole, with the potential to provide experiences that addict people’s low SOC^(10-11)^. GRDs, therefore, play a relevant role in people’s health. To the extent that they have the potential to weaken SOC, especially in those with weak SOC in early adulthood, they lead subjects into a vicious circle that weakens SOC, making life chaotic, uncontrollable and meaningless^(8)^. Therefore, when there is a lack of knowledge about GRDs and GRRs of a group of people, it is unlikely that it will be possible for health professionals to provide suggestions so that such subjects can strengthen their SOC. Thus, qualitative research can obtain knowledge about these people’s individualities, directing health promotion actions focused on their needs and also providing opportunities for them to manage stressors and create different life and health experiences^(12)^.

It is worth noting that, in the context of chronic diseases, a Brazilian integrative review on the use of SOC, the Salutogenic Theory’s key construct, concluded that nursing professionals use it in different groups of pathologies, in order to observe its relationship with objective and subjective health measures, with the Antonovsky Questionnaire correlating variables and scales. However, the predominant approach is quantitative instead of qualitative, which appears to be an open field for research^(13)^. Furthermore, a study carried out in Sweden, with elderly caregivers, found a greater number of GRDs in relation to GRRs, concluding that both can be drivers of health promotion actions at the individual and collective level as well as being able to direct health policies^(12)^.

Gap in knowledge on GRDs in the context of health in prisons is considered, since no studies were found that discussed GRDs in such a scenario in searches carried out in databases such as Virtual Health Library, PubMed and Scopus, which justifies the need to expand research on the topic and, in particular, based on the Salutogenesis framework. Thus, the relevance of this study is based on the understanding of Woodall *et al*.^(3)^ about the importance of PDL being able to express their experiences, opinions and needs and the absences with which they live in prison, which can direct future actions by prison health professionals and managers - a focus that is intended to be highlighted with this study, as PDL statements are made visible regarding the factors that make it difficult to maintain their health in prison.

## OBJECTIVE

To seize the GRDs of PDL with hypertension from a Brazilian prison unit.

## METHOD

### Ethical aspects

The study was conducted in accordance with national and international ethical guidelines, and approved by the Research Ethics Committee of the Health Sciences Sector of the *Universidade Federal do Paraná* (UFPR), following the recommendations of Resolution 466/2012, whose opinion is attached to this work. To guarantee participant anonymity, identifying codes were used to replace their names, such as P (participant), M (male) and X (Arabic number in ascending order of the participants interviewed) (e.g., MP_1). Furthermore, for employees of the penal system who were mentioned in the interviews, Arabic numbers were used in sequence, and all participants signed the Informed Consent Form (ICF).

### Study design

This is a descriptive and exploratory study, with a qualitative approach, based on the Salutogenic Model. This is part of a doctoral thesis project whose title is “*Doença crônica e saúde das Pessoas Privadas de Liberdade à luz da Teoria Salutogênica: estudo de métodos mistos*”. In this approach, it is possible, through its subjective nature, to dialogue, create meanings and meanings, capturing the phenomenon through the perceptions of the people who participate in the research^(14)^. To guide the research report, the COnsolidated criteria for REporting Qualitative research (COREQ) was used.

### Theoretical framework: Salutogenesis

The Salutogenic Model was developed by Aaron Antonovsky (1923-1994) in the 1970s and focuses on the origin of health: the central question is to understand how and why certain people remain well even after experiencing situations of intense stress and, even so, are located at the health-disease continuum healthy pole, despite stressors, and others are not. Thus, health or illness depend on the adequate management of tensions and the way in which people understand their lives^(4,8)^. Salutogenesis rejects the dichotomous health-disease classification, understanding it as a continuous process in which people are faced with the two poles throughout their lives - health and disease -, which are integrated into a multidimensional continuum. Therefore, people find themselves in progression or regression in relation to the health-disease poles^(15)^.

Therefore, human nature is understood as heterostatic, meaning that the human organism is in a dynamic state of imbalance; illness and internal and external stressors are the norm, inevitable; and non-homeostatic, with the organism regulated, balanced and occasionally disturbed by the disease, which seeks to control stressors, risk factors. Thus, chaos and stress are seen as part of life and the natural conditions of human living^(8,15)^. The search, from the salutogenic perspective, is not to determine whether an individual is healthy or sick, but to understand how close they are to the health-disease continuum poles and what are the factors that promote movement towards the healthy pole^(8)^.

### Study setting

The present study was carried out in a Brazilian prison unit, in a large city in the South region, which housed 614 male people over 18 years of age on the initial date of collection, from February to July 2022. The aforementioned penal unit had a small outpatient clinic for PDL health care, with the work of a multidisciplinary team (doctor, nurse, nursing technician and dentist) who monitored PDL’s health demands. During the research period, the team maintained all the penal unit’s usual precautions.

### Data source

The sampling used was for convenience, and used all PDL from the unit with a diagnosis of hypertension and who accepted participation (n= 38 or 6.2%). It was decided not to use saturation for sample delimitation, understanding that an ideal qualitative sample is one that reflects quantity, intensity and the phenomenon multiple dimensions, actions and interactions throughout the process^(16)^ that, for this study, was the delimitation of GRDs of PDL with hypertension. The inclusion criteria were being a PDL in the unit and having a diagnosis of hypertension.

Participant recruitment began in 2021 with the dissemination of research verbally and through folders in the penal unit. Subsequently, participants who met the inclusion criteria were invited to participate individually, at which time the research, objectives, usefulness and procedure were presented. The PDL who agreed to participate in the research signed the ICF and were instructed that non-participation would not cause them any disadvantage, and that they would maintain their standard treatments in the penal unit. One participant who met the inclusion criteria declined to participate.

### Data collection and organization

A semi-structured interview was used to collect data, as it is more flexible in nature and gives participants the opportunity to construct versions and meanings for the world in which they live, in addition to being possible for exchanges to take place between researcher and participant^(9)^. In this way, PDL were invited to speak freely about the topic, which was guided by the question: what are the factors that you consider make it difficult to maintain your health in the prison context? Thus, an instrument created by the researchers was used with 7 open-ended questions, using as frameworks the salutogenic model key concepts, such as SOC, GRRs and GRDs. It is evident that, for this study, we worked with excerpts that include the GRDs corresponding to the aforementioned question.

Interviews were carried out in an office in the prison health unit’s outpatient clinic by the first author, who is a nurse in a prison health unit. Therefore, they were audio recorded and transcribed*, a priori*, with Transkriptor^®^ and, afterwards, checked and organized by the researchers. Subsequently, data were arranged in a Microsoft Excel^®^ spreadsheet, with rows corresponding to participants and columns corresponding to the answers obtained in open-ended questions. The aforementioned spreadsheet was then imported into NVivo^®^ (version 12 - release 1.7 for Windows), which was used to store and organize the data. It should be noted that the software was used as an auxiliary resource to facilitate and organize the data analysis process, since it allows generating graphs, word clouds and word counts, automated coding, matrices, among others, but it does not replace the researcher and, therefore, it is not a data analysis modality.

In NVivo^®^, the main researcher read the material, automated analyzes provided by the program to direct coding (the software provides possible coding), and codes constitute the research analysis categories. Subsequently, exhaustive readings of reports were carried out, refinement and reorganization of codes or categories through thematic analysis. This study worked with codes/categories and subcodes/subcategories.

### Data analysis

Data were subjected to content analysis in thematic modality, following the steps relating to comprehensive and exhaustive reading, material exploration, and data treatment and interpretation^(17-18)^, initially being coded by the first author, with review and discussion by the research team. They then resulted in four categories: 1) Generalized Resistance Resources and Generalized Resistance Deficits; 2) Health, well-being and quality of life in prison; 3) (Lack of)Hope with life and the future; and 4) (Lack of) Health care. Furthermore, they generated ten subcategories: i) Factors that help maintain health in prison; ii) Factors that make it difficult to maintain health in prison; iii) Concept of health in prison; iv) Concept of quality of life in prison; v) Hopes for the future and life; vi) Hopelessness about the future and perception of life; vii) The search for maintaining health before prison; viii) The search for maintaining health in prison; ix) Sources of health information; x) (Lack of)Information. However, for this study, the “Generalized Resistance Resources and Generalized Resistance Deficits” category is highlighted only in relation to GRDs, included in the “Factors that make it difficult to maintain health in prison” subcategory.

## RESULTS

**Figure 1:**
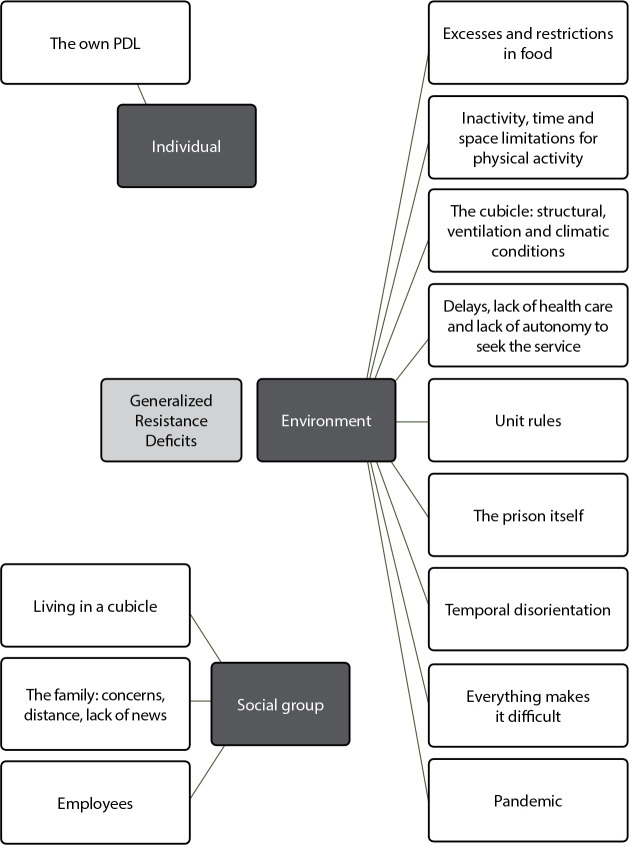
Generalized Resistance Deficits gathered in the reports of people arrested with hypertension. Foz do Iguaçu, Paraná, Brazil, 2023

A total of 38 male PDL participated in the study, 68.4% aged between 30 and 44 years, 50% married, 36.8% with between 9 and 12 years of education and 32.6% with less than 9 years of education. The total categorization of the *corpus* resulted in four categories and ten subcategories. For the present study, we worked with a subcategory included in the analysis of the “Generalized Resources of Resistance and Generalized Deficits of Resistance” category, with emphasis on the GRD “Factors that make it difficult to maintain health in prison”, nomenclature used in the guiding question for adequate and understandable language to participants at the time of the interviews. In the process of exhaustive reading of the material, speeches that dealt with limitations/absences for maintaining life and health in prison - GRDs were coded, resulting in 111 coded references (16.1%), listed by PDL in the total *corpus*. Figure 1 presents the GRDs found.

Below are the excerpts referred to and their respective descriptions.

### a) Excesses and restrictions in food

Food is described by PDL as a factor that makes it difficult to maintain health in prison due to: abundance and PDL’s impasse in maintaining a healthy diet, linked to the anxiety generated by the prison context; restriction on the quantity of food supply such as fruits and vegetables; and frequent supply of processed foods, such as breaded and sausages and restriction of food entering the unit.

*Look, what makes it difficult would be the ease we have at the moment. The issue with food is access. If you have a little more access, you will eat more, especially since we are deprived of freedom, which sometimes makes us a little anxious.* (MP_1)

*Which makes it difficult, just like those fruits that don’t come, that only come once a week and I think that’s it. So, look at me, I think it is. It comes once a week; I also don’t know exactly how much it came this week. So, having it every day is already difficult. Sometimes it doesn’t even come, it takes a while.* (MP_20)

### b) Inactivity, time and space limitations for physical activity

According to PDL, the absence of activities is an element that makes it difficult to maintain their health, since they remain idle for a long time and restricted to the environment of cubicles (cells, which are very small compartments similar to a room where PDL), situation that they themselves present as generating physical and psychological illness. The routine of deprivation of liberty, for some PDL, is characterized by the lack of occupations and/or objectives and, therefore, they use a large part of the time to sleep and/or reflect on life; reports the difficulty in carrying out activities in a small place, a cubicle, which is shared with 5-8 people as well as highlighting the difficulty in being directed to study and/or work activities; some PDL already carry out such activities and indicate a minimum amount of time reserved for them.

[...] *it’s idle* [...] *that’s what makes it more difficult. A lot of free time* [...] *I struggle a little to sleep, especially without having activities like that, because my mind is very empty, then one day I sleep well, and two or three days pass without sleeping, it gives me a bit of a headache and so it goes. Don’t get tired, the body is always rested.* (MP_35)

*I’m doing crafts and reviews. There are other activities, but the review only takes place one day and they pick it up the next day. And there’s little crafts too. It’s quick to do. It’s just that. I’ve already made a couple of things for us to try as a bricklayer, a carpenter to work with, but so far, nothing yet.* (MP_30)

PDL pointed out limitations to physical activities related to time available to them on the sun patio, which varies between reports of 40 minutes to 5 hours a week, being canceled on rainy days and/or when there are other activities in the penal unit. They also point to the limited space of the place, which limits the possibilities of different types of physical exercise as well as the high number of people in the small space.

*The psychological problem, that’s it, there’s this issue, the fact that an hour on the playground is five hours a week, so there isn’t much time left for practicing sports, so, as I said, there’s no way to do one exercise, at least for walking.* (MP_32)

[...] *in the courtyard, it’s also a short time, it’s one hour a day, four hours a week, and sometimes there isn’t.* (MP_13)

The situation of imprisonment, idleness and the treatment reserved for PDL generate, at times, the “desire to be worse”, and the support to face such a situation is related to “thinking about life” and their families. They feel, at certain times, in the same and/or worse conditions than those reserved for animals.

[...] *we were in a cubicle there three by three to five, six people, you feel like an animal in confinement* [...]. (MP_14)

[...] *because of the situation I am subjected to, I want to be worse than I was before on the street* [...] *we see on television. He left the dog locked inside the car with just a little bit of wind, poor dog, poor thing, I don’t know what, so I’m treated worse than an animal then, because I’m locked out of this thing and I don’t have ventilation* [...]. (MP_32)

### c) The cubicle: structural, ventilation and climatic conditions

The cubicle’s structural conditions were highlighted by PDL as elements that make it difficult to maintain health related to: insufficient space for all people to coexist (some pointing out five and others eight people in the same cubicle, related to differences in the number of people between the unit galleries, which is made up of the main building and the annex), in particular making it difficult to practice physical exercises in this location; insufficient ventilation, with extreme heat during summer and cold in winter - which is related to the lack of blankets and clothing for heating and fans that cannot be turned off in the cell; sharing bedroom/bathroom space; fully closed door that makes it impossible to see outside; and place ventilation.

*Which perhaps makes a space difficult. We don’t have much space here. Yes, the cell is small! The deal is five-four. So, for instance, if, now it’s my time, let’s say I do an exercise and such, there’s no way everyone is going to do it at the same time, there’s no way! We stayed closed for a long time.* (MP_11)

[...] *this unit here was a unit that it was not, it was not planned* [...] *because it ends, it ends up stressing inmates and even employees.* [...] *besides walking, there’s no way, there’s no way, because there’s no way to do it here.* [...] *it’s a unit where you have to wash the shack, wash the shack as we say, the shack, the cell, then you have to remove all the water. For me, that there is good, for me, I’m doing some physics, you know, but there’s no way for water to drain, there’s no drain for it to drain* [...], *but, as we have this difficulty, the space is little* [...]. (MP_13)

### d) Living in a cubicle

Living with cubicle mates was also presented by some PDL as an element that makes it difficult to stay healthy, related to the stress that living with so many people in a small, closed place can generate and/or with the possibility of resulting in physical attacks and illnesses.

[...] *there are a lot of fights, a lot of fistfights, because then a fight can be an argument that never knows where it will end, that’s where it interferes. Myself, me, my blood pressure, it changed a lot because of the anger of a caboclo I lived with* [...]. (MP_26)

*It’s daily coexistence. On the street, we live with someone and, if we don’t get along very well, we leave, go to another corner, and in jail, the way we are, even if we don’t like the person, we have to live together all day, so there are times when it stresses us out.* [...] *coexistence is stressful.* (MP_31)

### e) Delays, lack of health care and lack of autonomy to seek the service

The delay in accessing health care was another element that makes it difficult to maintain health, with health teams in insufficient numbers; high number of PDL attached to the team; lack of autonomy to access the health service, since, to travel to the unit’s outpatient clinic, PDL need help from the “gallery cleaner” (nomenclature directed to another PDL that works in galleries and provides information about inmates’ needs) and, in particular, from security team employees, who need to comply with all safety procedures and routines for subsequent transfer of PDL to health care: activation of internal control employee (who opens doors); opening the cubicle door; opening the gallery door; user magazine; and displacement to the health sector. This difficulty is accentuated on days and times when the health team, which works on a daytime outpatient basis, is not at the unit, requiring, in addition to the procedures mentioned above, the request for escort and activation of the municipal network’s Mobile Emergency Care Service as well as services that require referrals to the municipal health network due to the need to activate the escort.

*It’s more difficult, it’s a little more difficult because of being trapped. Ah, it makes it difficult because sometimes he can’t get an appointment. They take time, sometimes we are feeling something that we don’t know what it is, there’s no way to ask right away. I can’t, come to the appointment alone, only if the guards bring it, ask, you have to make an appointment and it takes a while. To bring it, yes, you always need someone.* (MP_27)

*But I understand what they say. But then it always takes a while because, for example, today, I have high blood pressure. They will see me tomorrow or the day after, tomorrow or the day after, everything has changed. That’s when you can’t know more, you can’t control it.* (MP_35)

### f) The family: concerns, distance, lack of news

Concerns about the family, especially wives and children, mainly related to lack of news from family members, were presented as elements that generate stress, depression and repercussions on physical health by PDL, as they worry about their health, financial conditions, food, bonding with family, among others. They point out the distance from their family members as one of the elements that make it difficult for family members to visit them, a fact that can be understood, since the unit studied brings together inmates from a certain criminal faction from all over the state.

*Here? I think I’m going to tell you that I think so, that there are factors that make it difficult. Because for many, for many here, it is worry, everything that is happening, and those who are far away have no news about their family, and concern takes away not only your attention but also the increase in concern.* (MP_16)

*It is hard. Being away from my family is making it difficult for me, it’s slowly killing me* [...] *because you think that a lot of things, you’re eating something, but you think, “Are they eating? Are they okay? What are they doing? Are they going to school?”. There are times when I can’t even sleep because of it.* [...] *being far away hurts because I’m not even the pillar of my family, and this one affects me a lot because I think too much and it often changes the person’s mood, and emotional things often affect a person’s emotions.* (MP_17)

### g) Unit rules

The unit rules, routines and safety procedures, according to PDL reports, constitute limitations for maintaining health, as they cannot use weights in the cubicle to carry out physical activities, such as restricting the entry of certain foods and medications, in addition to bathing only once a day and the oppression.

*Lots of things that make it difficult. Ah, the very oppression that inmates suffer is what most leads inmates to have health problems, because, let’s suppose, there is an employee who arrives and out of nowhere he applies that pepper spray* [...]. *So, I calculate that this has a lot to do with our health.* (MP_14)

*In the cell, you can’t have anything, you can’t have anything, you can’t have a weight tied up, you can’t have anything, nothing, nothing, nothing. So, you have to adapt to what is inside the cell.* [...] *here we can’t do it anymore, here medicine, things to keep us healthy, like that, even in itself like that, fruit, those things, I at least think it helps our health.* (MP_24)

### h) The prison itself

The condition of imprisonment and absence of freedom make health impossible, as they generate stress, anxiety and suffering, described as a bad feeling.

[...] *in fact, it’s being in prison that makes our health difficult, that we get stressed, but there’s nothing we can do, because we committed a crime, we made a mistake and we have to pay, right? It’s about being without freedom. It’s true. Oh, you get upset, you get anxious, you get angry because you’re paying so much jail time for a crime that you, like I wasn’t even talking about today, wow, if I had known that cigarettes would get you so much jail time, I wouldn’t have made the mistake.* (MP_21)

*What makes it difficult to be in this place, right? You suffer a little, you suffer a little being in here. The feeling of being trapped is bad. Ah, you feel like you can’t be close to your family, that you can’t do anything.* (MP_31)

### i) Employees

A participant reported that, at times, they are disrespected verbally with humiliation and insults and, physically, with pepper spray and rubber bullets.

[...] *we try to be polite, and I often try, at least in my daily life, to be a polite person* [...] *treat employees with respect, knowing how to talk to them or feel threatened either and often also who would have to treat me with respect* [...] *sometimes, we are humiliated, we go through different situations that other human beings sometimes cannot understand why we are subjected to a situation there. I hear stories about people who sometimes go to steal there and even in a certain way have a certain respect, a certain limit, often, for no apparent reason, it’s pepper spray, rubber bullets, situations that are inhumane, that’s complicated.* (MP_32)

### j) Temporal disorientation

MP_35 also pointed out temporal disorientation as an element that makes it difficult for him to stay healthy.

*That’s what makes it difficult, the lack of guidance over time, you get lost, you lie down a lot, it makes it very difficult to take care of your health.* (MP_35)

### k) The person deprived of liberty

MP_15, MP_19 and MP_38 attributed the difficulty in maintaining their health to themselves as they did not adhere to drug and non-drug treatment.

*Here inside. Look, the only thing that makes it difficult, I’m making it difficult myself, by not taking my medication, by not taking care of myself.* (MP_15)

*I don’t think there’s anything that makes it difficult. What makes it difficult is myself. It’s because, sometimes, we don’t want to take up other people’s space. We’re going to stay quiet in our corner so as not to disturb others, right, we’re not alone, there are 8 of us in the cell. I try not to disturb anyone so that no one can see me.* (MP_38)

### l) Everything makes it difficult

For MP_16 and MP_24, everything in the prison environment harms the maintenance of their health, since nothing helps them.

*I believe that nothing is helping me to take care of my health.* (MP_16)

*Ah, inside the prison, at least here, it doesn’t give me anything.* (MP_24)

### m) Pandemic

The context of the coronavirus pandemic was presented by MP_33 as aggravating health maintenance as it made contact with family members difficult.

*And now it’s gotten more complicated because they miss me a lot, it’s a lot. With this pandemic, it is difficult, as the visit is once a month, so it becomes very complicated.* (MP_33)

## DISCUSSION

This study was designed to present GRDs among PDL with hypertension, an essential concept for the health and life management process, since GRDs can weaken SOC, determining the person’s movement in the health-disease continuum. It should be noted that Salutogenic Theory has been identified as an adequate theoretical framework for health promotion, and can be used in planning health actions and policies also for PDL. However, it is worth highlighting that knowledge about GRDs is still scarce^(12)^, especially in imprisoned populations.

It is considered that, from the reports, 13 GRDs emerged, lack of resources or factors that make it difficult to maintain health in prison, which were related to individuals (self-care), social group (other inmates, penal and health system employees) and environment (structures, services and routines in prison). Therefore, the high number of GRDs seized is highlighted, which highlights the difficulty of maintaining health in prison, since when GRDs are insufficient, combating stressors becomes more difficult^(19)^.

It is worth noting that GRDs, or the absence of GRRs, interfere with the person’s movement along the health-illness continuum, with the possibility and ability to manage the tensions inherent to life and migrate to the health pole^(2)^. Therefore, for PDL, movement in the continuum presents a high number of stressors, therefore, reflecting Antonovsky’s River of Life metaphor. These people are at the forks of the river that lead them to the rapids (which represent danger). For those close to the waterfall, the fight for survival is more difficult^(7)^. However, knowing such GRDs can lead to health-promoting actions in order to minimize them and/or support GRDs.

In this sense, given the GRDs seized and because they are mostly related to the environment, it is reiterated that, despite the legal delimitation, health care in prison needs to be equivalent to that provided in the community^(1)^. This study establishes relationships of conclusions with other Brazilian studies, such as the research carried out in Minas Gerais, which pointed out the right to health is still perceived as a norm that does not materialize in PDL’s daily lives^(20)^, as well as being aligned with the study with women inmates, which highlights the principle of equality as historically subjugated in prisons^(2)^.

Furthermore, the reports of participants in the present study point to delay, absence of health care and lack of autonomy in seeking health services, corroborating the Italian qualitative study with 10 PDL, which showed that the health system in those prisons did not meet the needs of the people imprisoned there^(21)^. Furthermore, the present data are also included in another study with Brazilian PDL, which found barriers to the inclusion of such subjects in the Health Care Network^(22)^.

It is also in line with the Swiss qualitative study, which interviewed 35 PDL over 60 years old, which highlighted situations that were harmful to PDL’s health: limitations in infrastructure; cell structures that did not adapt to the specific health needs and the health unit itself; schedules; security measures; shortages of certain medical equipment and treatments; and limitation of autonomy for access to services^(23)^. Thus, PDL find themselves constrained in positions of submission to a hierarchical disciplinary order, since they depend on the security team for their referral to the health sector^(20)^.

As in the aforementioned study^(23)^, this research demonstrated that prison can limit access to health care related to the prison health service opening hours, which may not be available full-time. As in the unit of this investigation, the Swiss prison analyzed by the authors also had daytime health services only, which implied demonstration by PDL of the need to activate the emergency service at night, submission to bureaucracy, the possibility of non-attendance and the need for logistics from the security team to transport PDL to health care. Swiss PDL also expressed that consultations outside the prison caused them discomfort related to transportation in prison clothing and/or handcuffs^(23)^.

With regard to social ties, the conflicting relationship with security team employees in relation to verbal and physical disrespect. PDL’s statements in this research corroborate a study with 35 PDL in the United Kingdom, which obtained reports that prison officers were not concerned with inmates’ health and well-being, lived with low interaction, and had their requests ignored and devalued^(24)^. It is emphasized that severe conditions of imprisonment do not reduce the likelihood of recidivism, but may result in an increase in post-release criminal activities^(21)^: situation explained in the testimony of a participant in this research, who pointed out the desire to “be worse” due to the situations he is subjected to.

In relation to the difficulties of coexistence with other PDL in cubicles, reported by the participants of this research, which generate physical and verbal insults, they are in line with reports of aggressions from other inmates that were also narrated by PDL from the United Kingdom^(24)^.

Regarding relationships with family members, reports indicated that the absence of news from family members and/or visitors negatively affected their health. Therefore, social relationships, especially with the family, are closely related to the concept of being healthy for PDL. Contact with the family is central to their values, as it restores personal dignity, positively impacting their rehabilitation and reintegration into society, including the potential to prevent criminal recidivism^(21,25-26)^.

Living in prison, for PDL not included in work and/or study activities, is routine with long periods of idleness in cubicles and, thus, they live without occupations and/or goals, using much of their time to sleep and/or or reflect on their lives, as they only have activities outside the cell for about 40-60 minutes a day. Then, in their speeches, they highlighted the need for more time outside the cell, access to open air and natural light to feel well as well as highlighting the difficulty they encounter in engaging in study and/or work activities. Those who have already been deployed in such occupations explained a minimum amount of time reserved for these activities. In contrast to the reports is the legal provision, explained in the Criminal Execution Law (CEL)^(27)^, which is insufficient to guarantee PDL access to rights such as education, health and work.

This research is corroborated by an ethnographic study carried out with 500 male PDL in the United Kingdom, which concluded that they were forced into long periods of idleness and/or underemployment in prison. PDL complained of idleness, apathy, boredom, lack of motivation, lack of limited and rationed education and training opportunities^(24)^. Furthermore, the study with Italian PDL found reports that education and knowledge in the perceptions of PDL could even increase the confidence of inmates in institutions and in society as a whole, since occupations provide them with existential motivation to become better people^(21)^. A study carried out in South Korea stands out, which showed that horticulture resulted in a reduction in depression, increased self-esteem and satisfaction with life^(28)^.

Another GRD reported by PDL in the research presented here was the limitation of time and space for physical activity, which makes it difficult to maintain their health in prison. In a Swiss study with 152 PDL, it was concluded that physical activities reduce psychological and psychiatric suffering, anxiety and depression in PDL^(29)^.

Furthermore, another element presented as GRD was food in the penal unit. Although planned by a nutritionist, its abundance was aligned with PDL’s difficulty in maintaining a healthy diet, along with the anxiety generated by the prison context; restriction on the quantity of food supply such as fruits and vegetables; frequent supply of processed foods, such as breading and sausages; and restriction of external food entering the unit. All these situations corroborate the studies with 25 PDL from the state of Rio de Janeiro, in Brazil, in the sense of low nutritional quality, diverging with reports of tasteless, spoiled food, with impurities, insects and periods of hunger^(30)^. Thus, the situation concerns a Mexican study that concluded that prison exposed PDLs to low-quality diets^(31)^. Thus, even though legislation guarantees them food with sufficient nutritional value for health and physical vigor, the right does not seem to be covered in prisons.

Considering the GRDs discussed, it is considered that incarceration in itself is harmful to health, even if the statistics do not capture the experiences lived in prison and even if they demonstrate lower mortality among PDL than in the community. Living in prison implies health problems related to confinement that can be maximized by poor housing conditions, inadequate food, poverty, discrimination, loss/lack of support networks and control over their lives^(32)^, as the present study shows by the high number of GRDs, directing PDL to the disease pole. Thus, health inequities in this population arise from marginalization of PDL in access to health before deprivation of liberty related to social vulnerability, high rates of physical and mental illness, abuse of illicit substances and alcohol. These situations continue and worsen with restricted access to prison health services and unsanitary conditions in prisons^(33-34)^.

Therefore, prison health needs to reflect on PDL’s health, their experiences, in addition to understanding the prison context, its physical (environment) and organizational structures, relating such factors to the social determinants of health that permeate the prison setting, in constant dialogue with society in general, in order to create better living and health conditions for this population group^(2,24)^. Knowing the GRDs of such a population group makes it possible to direct actions to these people’s demands. It is highlighted that better prison structures, recreation, productive occupation, reduction of abuse and extinction of violence are indirect mechanisms that can promote health and result in fewer needs to activate the health service^(35)^.

Knowing the GRDs, then, allows health professionals to direct health care planning to the PDL group’s needs, filling the gaps and absences that are explained by the PDL themselves. Thus, it provides opportunities for the health team to manage the health-disease continuum with a view to strengthening GRRs and minimizing GRDs in a continuous process of advocating for better living and health conditions for PDLs. Furthermore, it provides support to prison health managers and the prison itself in the search for improvements in structures, routines and work processes, aiming to minimize GRDs.

### Study limitations

The limitations of this study are related to lack of research on the topic, making it difficult to discuss the results. However, it is asserted that this fact highlights the originality of the theme.

### Contributions to nursing, health and public policies

The findings of this research expand the scope of knowledge about health in prisons from a salutogenic perspective, providing support for the development of prison health policies based on absences perceived by PDL. In care practice, they can direct health professionals to actions that promote GRRs, with a view to strengthening SOC for better life and health management conditions for PDL.

## FINAL CONSIDERATIONS

A high number of GRDs were detected in the reports of PDL with hypertension, with a predominance of those related to the prison environment and, to a lesser extent, to the social group and the individual, respectively. Therefore, possibilities for effective management of their lives and health are limited, with greater chances of migrating towards the disease pole. The need for health-promoting actions with PDL is evident, in individual and collective approaches, through intersectoral policies to support the use/availability of GRRs, especially related to the prison environment, which is shown to increase illness.

This study reveals the relevance of understanding the GRDs present in a group of people, and asserts that such knowledge allows the planning of health-promoting actions centered on the population group’s needs. The salutogenic approach and GRDs are emphasized, among other concepts, as fundamental to direct policies for Primary Health Care.

## Data Availability

*https://doi.org/10.48331/scielodata.SCQYEN*
